# Reduced Risks of Both Ischemic and Hemorrhagic Strokes in Nurses: A Population-Based Cohort Study in Taiwan

**DOI:** 10.3390/ijerph15122615

**Published:** 2018-11-22

**Authors:** Hui-Chuan Liao, Yi-Hao Peng, Yu-Pei Chen, Li-Chi Huang, Wen-Miin Liang, Chung Y. Hsu, Chia-Hung Kao, Chun-Hung Tseng

**Affiliations:** 1 Department of Public Health, China Medical University, Taichung 40447, Taiwan; n5929@mail.cmuh.org.tw (H.-C.L.); rich79395@yahoo.com.tw (Y.-H.P.); 2 Department of Nursing, China Medical University Hospital, Taichung 40447, Taiwan; lichi@mail.cmu.edu.tw; 3 Department of Respiratory Therapy, Asia University Hospital, Asia University, Taichung 40447, Taiwan; 4 Department of Respiratory Therapy, China Medical University, Taichung 40447, Taiwan; 5 Management Office for Health Data, China Medical University Hospital, Taichung 40447, Taiwan; baychen201710@gmail.com; 6 College of Medicine, China Medical University, Taichung 40447, Taiwan; 7 School of Nursing, China Medical University, Taichung 40447, Taiwan; 8 Center for Faculty Development, China Medical University, Taichung 40447, Taiwan; wmliang@mail.cmu.edu.tw; 9 Graduate Institute of Clinical Medical Science, China Medical University, Taichung 40447, Taiwan; hsuc@mail.cmuh.org.tw; 10 Graduate Institute of Clinical Medical Science and School of Medicine, College of Medicine, China Medical University, Taichung 40447, Taiwan; d10040@mail.cmuh.org.tw; 11 Department of Nuclear Medicine and PET Center, China Medical University, Taichung 40447, Taiwan; 12 Department of Bioinformatics and Medical Engineering, Asia University, Taichung 40447, Taiwan; 13 Department of Neurology, China Medical University Hospital, Taichung 40447, Taiwan

**Keywords:** ischemic stroke, hemorrhagic stroke, nurse, cohort study

## Abstract

Background: Nurses are typically required to address patient emergencies, and they experience high stress levels in their work, which may expose them to a higher risk of stroke. This cohort study compared the risk of stroke between nurses and the general population. Methods: We used the Taiwan National Health Insurance database to conduct our retrospective cohort study, and we identified 83,641 individuals in the nurse group and 334,564 individuals in the control group. For the nurse group and the control group, we used the chi-square test in addition to applying Student’s *t*-test, in order to compare the distribution differences for the continuous variables. We estimated the hazard ratios (HRs) and 95% confidence intervals (CIs) for ischemic stroke and hemorrhagic stroke through univariate and multivariate Cox proportional-hazards regression models, with stratification according to age, sex, and comorbidity. Results: The nurse group had a lower risk of ischemic stroke and hemorrhagic stroke in the crude model (HR = 0.66, 95% CI = 0.58–0.75; HR = 0.58, 95% CI = 0.47–0.72). After adjusting the prevalent variables, the nurse group still had a lower risk of stroke (HR = 0.68, 95% CI = 0.60–0.77; HR = 0.59, 95% CI = 0.48–0.73). Conclusion: The risks of both stroke types were lower in the nurse group than in the control. For stroke prevention, more frequent physical examinations are needed in order to enhance the health and well-being of people, including the nurses.

## 1. Introduction

In general, nurses have to address patients’ emergencies, and it has been reported that 50% have expressed concern regarding high occupational stress [1,2,3,4]. The circadian rhythm and lifestyles of nurses’ are disrupted by their rotational work shifts, and this disruption is a major risk factor to injure their health [5]. The National Health Insurance (NHI) program in Taiwan was implemented in 1995, and has covered 99% of the residents of Taiwan. This program plays an essential role in promoting the health of Taiwanese people. However, the favorable coverage provided by the NHI program has engendered an increase in medical needs, and thus imposed a higher workload on healthcare practitioners in Taiwan than those in other countries. For example, a nurse in Taiwan cares, on average, for 10 to 15 patients at a time. This is two to three times the number for every nurse in Europe, the United States, Australia, and Japan—and each nurse in Taiwan works for 10 to 12 h per day [6,7].

Long-term exposure to long working hours and high occupational stress expedites cellular senescence and increases the risk of early death [8], and, in addition, this is also significantly correlated with the development of high blood pressure [7,9,10,11], stroke, and cardiovascular disease (CVD). A study demonstrated that a high workload and high occupational stress are major risk factors for stroke [12]. Overall, the nursing profession is highly stressful, and addressing patients’ emergencies for a prolonged period is detrimental to health. These problems are not only prevalent in Taiwan, but are also observed among young nurses in the United States and European countries [13,14]. Hence, numerous future nurses are hesitant to enter the workplace. This has led to the phenomenon of an aging workforce and late retirement among nursing personnel. Countries worldwide are facing the challenges of a lack of nurses, and have to compensate more for this. This exerts negative effects on the health care industry.

To the best of our knowledge, there were only a few studies investigating the correlation between the nursing profession and the risk of stroke development. Therefore, the purpose of the current study was to measure the risk of stroke among nurses and the general population (controls), using a population-based cohort. We hypothesized that nurses, who face high occupational stress and a high workload, are at a higher risk of stroke than the controls. The findings of this study can provide the implications needed to enhance the health of the nurses themselves, and of the personnel who work in the government health agencies. On the basis of the findings, more effective policies and more friendly working environments can be created in order to improve nurses’ health condition, and these could enable them to provide more sustainable care for their patients.

## 2. Materials and Methods

### 2.1. Data Source

We used the database of the Taiwan NHI program to conduct our retrospective cohort study. The NHI program was implemented in March 1995 and provides coverage for up to 99% of the 23.74 million residents in Taiwan; its database contains information about these individuals. We determined the disease history of the individuals from the files of inpatients claims and from the Registry of Beneficiaries. All of the diagnoses and disease definitions in our study were specified by the International Classification of Diseases, Ninth Revision, Clinical Modification (ICD-9-CM) codes. The study was approved by the Research Ethics Committee of China Medical University and Hospital in Taiwan (CMUH104-REC2-115-CR2).

### 2.2. Identification of Nurses and Selection of Controls

For the nurse group, we obtained the data on the nurses from the Registry of Medical Personnel, which contains the records of all of the registered medical staff members actively employed in Taiwan since a year before 2000 or in 2000. All of the nurses with license registrations before or in the year 2000, who were actively employed as nurses by 31st December 2000 (index date) were enrolled. For the control group, we selected four nonmedical staff matches per nurse from the Longitudinal Health Insurance Database 2000, which contains all of the claims’ data of 1 million (4.34% of the total population) beneficiaries who were randomly selected in 2000.

**Figure 1:**
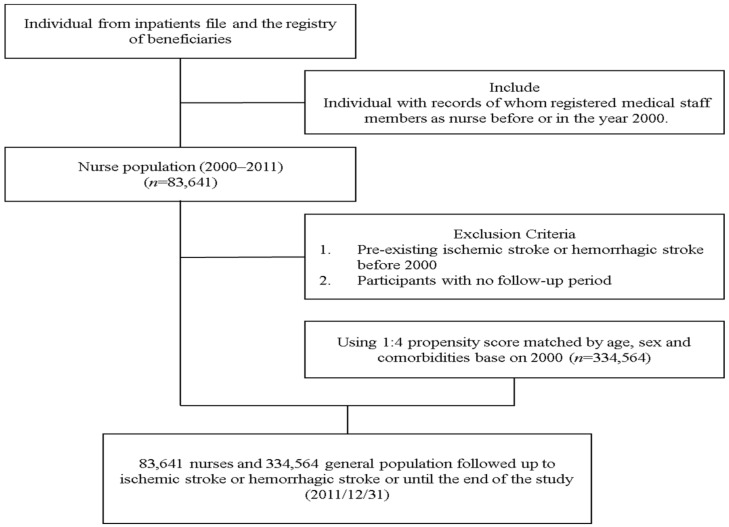
Flowchart for the selection of study individuals.

We matched the controls with nurses through frequency matching for age (every five-year span), sex, and comorbidities at the index date. The study period was the duration between the index date and the study endpoint, which was defined as the occurrence of ischemic of hemorrhagic stroke, withdrawal of health insurance, death, or the end of 2011. The individuals with a previous diagnosis of ischemic stroke (ICD-9-CM codes 433–438, A292, 293, 294, 299) or hemorrhagic stroke (ICD-9-CM codes 430–432, A290, 291) were excluded (Figure 1).

### 2.3. Identification of Ischemic Stroke and Hemorrhagic Stroke Cases

We followed the nurse and the control groups until the study endpoint (onset of ischemic or hemorrhagic stroke, fatal, or non-fatal), withdrawal of health insurance, death, or the end of 2011. The baseline comorbidity histories of hypertension (ICD-9-CM codes 401–405), diabetes (ICD-9-CM code 250), hyperlipidemia (ICD-9-CM code 272), coronary artery disease (CAD) (ICD-9-CM codes 410–414.02), CVD (ICD-9-CM codes 390–429), chronic kidney disease (CKD) (ICD-9-CM code 585), chronic obstructive pulmonary disease (COPD) (ICD-9-CM codes 491, 492, 496), heart failure (ICD-9-CM codes 428), obesity (ICD-9-CM codes 278.00, 278.01, 278.02, and 278.1), asthma (ICD-9-CM codes 493), and lung cancer (ICD-9-CM codes 140–208) were identified according to the patients’ diagnoses in the inpatient claims data prior to the index date.

### 2.4. Statistical Analysis

We compared the demographic factors, including age, sex, and comorbidity, between the nurse group and the control group using a chi-square test, as well as the Student’s *t*-test, to compare the distribution differences for the continuous variables. 

We estimated the hazard ratios (HRs) and the 95% confidence intervals (CIs) for ischemic stroke and hemorrhagic stroke by using univariate and multivariate Cox proportional-hazards regression models, with stratifications based on age, sex, and comorbidity. We adjusted the variables of age, sex, and comorbidities in the multivariable models. SAS 9.4 for Windows (SAS Institute, Cary, NC, USA) was used for all of the analyses. The significance thresholds were considered at a two-sided *p* value of 0.05.

## 3. Results

In this matched cohort study, we identified 83,641 individuals in the nurse group and 334,564 individuals in the control group with similar distributions in age, sex, and comorbidities (Table 1).

During a median follow-up after 11 years, we observed a total of 1937 ischemic stroke events (285 in the nurses and 1652 in the controls). Table 2 presents the crude HRs (cHRs) and adjusted HRs (aHRs) of the risk factors for ischemic stroke. Older individuals and individuals with any of the comorbidities had a higher risk of ischemic stroke (*p* < 0.001 for all). Regardless of sex, we observed no significant difference in the risk of ischemic stroke between the groups.

During a follow-up, we observed a total of 753 hemorrhagic stroke events (99 in the nurses and 654 in the controls). Table 3 presents the cHRs and aHRs of the risk factors for hemorrhagic stroke. Older individuals and individuals with any of the comorbidities (except for COPD) had a higher risk of hemorrhagic stroke (*p* < 0.001 for all). Regardless of sex, we observed no significant difference in the risk of hemorrhagic stroke between the groups.

The incidence rates of ischemic stroke and hemorrhagic stroke for both groups were stratified by age, sex, and comorbidity (Table 4). The overall incidence density of ischemic stroke was significantly lower in the nurse group than in the control group (0.32 vs. 0.48 per 10,000 person–year, cHR = 0.66, 95% CI = 0.58–0.75), with the corresponding aHR being 0.68 (95% CI = 0.60–0.77). The nurses had a lower risk of ischemic stroke in all of the stratified groups, except for the groups of male individuals and individuals aged younger than 35 years (with or without model adjustment). The overall incidence density of hemorrhagic stroke was significantly lower in the nurse group than in the control group (0.11 vs. 0.19 per 10,000 person–year, cHR = 0.58, 95% CI = 0.47–0.72), with the corresponding aHR being 0.59 (95% CI = 0.48–0.73). The nurses had a lower risk of hemorrhagic stroke in all of the stratified groups, except for the groups of male individuals, individuals aged younger than 35 years, and patients with any of comorbidities (with or without model adjustment).

## 4. Discussion

The high stress levels and long working hours that are associated with the nursing profession have been a long-term concern in Taiwan. This raises the question as to whether high stress levels increase the risk of stroke in people. However, after searching PubMed using key terms such as “nurses”, “stroke”, “risk”, and “cerebrovascular disease”, we could not find studies investigating the risk of stroke in nurses. This paper is thus the first to conduct a population-based cohort study with nurses as the main subjects. Specifically, we compared the risk of stroke between nurses and controls. Our finding indicates that the risk of stroke was lower in nurses than in the controls, which is consistent with the finding that physicians have a lower risk of stroke than controls [15]. Studies have reported that although health professionals face long working hours and high stress levels, their extensive medical knowledge and higher level of cooperation with treatment recommendations enables them to effectively manage high blood pressure and hyperlipidemia, thus preventing these chronic diseases from advancing to stroke. Another study compared the risk of diabetes between nurses and controls, and also found the risk to be lower in nurses. In this study, we found that the risks of both ischemic and hemorrhagic strokes were not significantly different between the nurse and control groups. In the age 35–50 and >50 groups, the risks of both stroke types are lower in the nurse cohorts. Although the older nurse subgroups had lower risks of both stroke types than their counterparts, the risks still increased with aging. This could be because the nurses receive medical education and training, and thus have knowledge regarding the prevention of chronic diseases [15,16]; they can apply such knowledge to adjust their lifestyles in order to prevent diseases and reduce the death rate from such diseases. Furthermore, studies have reported consistent results that the risk of chronic diseases was lower in health care professionals [15,16,17,18,19]. These findings show that extensive medical knowledge is beneficial to personal well-being and is helpful in disease prevention. Nevertheless, not all of the studies have revealed a lower risk of diseases in health care professionals. For example, a study conducted in 2015 suggested that the risk of migraine was higher in nurses than in controls, and that the risk was highest among health care professionals [20]. The higher risk of migraine among nurses could primarily be attributed to the need to address patient emergencies, heavy workload, high stress levels, and sleep dysfunctions caused by rotating shifts [20]. Studies related to the epidemiology of peptic ulcer disease have also reported a higher risk of this disease in nurses than in controls [21]. The inconsistencies between the findings of these studies and those of the present study could be because nurses work rotating shifts, experience high stress levels, and are the first-line personnel to address patients’ complaints and health condition variations.

Overall, the reasons for the lower risk of stroke among nurses despite their long working hours and highly stressful work can be categorized into four categories. (1) Nurses receive medical education and have a higher rate of health-seeking behavior when they discover any anomalies in their bodies. They also commonly maintain healthy lifestyles, thereby promoting personal well-being and lowering the risk of stroke [15,16,17,21]. (2) Nurses also enjoy convenient access to medical care in the event of disease incidence [22,23,24]. This enables them to receive immediate medical treatment and to achieve effective cooperation with continued treatment, preventing diseases that may lead to stroke [25]. (3) Smoking is prohibited in hospitals throughout Taiwan; therefore, the smoke-free working environment of the nurses could be another reason for their lower stroke risk [15]. (4) From a policy perspective, the “Regulations of the Labor Health Protection” implemented by the Taiwanese government requires a health examination every five years for personnel aged younger than 40 years. Public health institutions in Taiwan require health professionals to receive a follow-up X-ray each year in order to ensure optimal health protection, and many hospitals provide annual physical examinations for their medical staff members. Additionally, in the Taiwanese hospital accreditation system, a hospital staff health check is included as a bonus item for promoting health in hospitals. Many hospitals provide annual physical examinations for nurses and follow up on high risk factors for stroke, such as high blood pressure, hyperglycemia, hyperglycemia, and other diseases, through long-term health monitoring. 

## 5. Limitations

The National Health Insurance Research Database (NHIRD) does not provide information about the demographics and lifestyles of patients, such as their education level, diet, smoking status, and exercise habits. However, differences in these factors may increase the risk of stroke. Therefore, we considered risk factors such as high blood hyperglycemia, hyperglycemia, DM, CAD, obesity, COPD, asthma, and lung cancer, in order to further verify the risk factors for stroke among nurses. We could not obtain data regarding the working hours or the level of stress at work, and the correlation between stroke and high stress levels and that between stroke and long working hours remains unclear. Future research could elucidate this correlation.

## 6. Conclusions

In this matched cohort study, we used the data from the NHIRD to compare the risk of ischemic and hemorrhagic stroke, between nurses and controls. The results indicate a lower risk of ischemic stroke and hemorrhagic stroke in nurses than in the controls; our sample size was sufficiently large to be representative of the population of Taiwan, and the adjusted odds ratio was 0.68 and 0.59, which was significantly different between the nurses and the controls. Compared with the controls, perhaps because nurses have higher levels of medical knowledge, they can apply such knowledge to maintain healthy lifestyles, which may help reduce the risk of stroke. Moreover, the frequency of health examination is higher in health care professionals than in the controls, and this might be one of the main reasons for preventing the advancement of chronic diseases to stroke. The study has implications for health care policies in Taiwan. Specifically, health care institutions may benefit from educating the population about stroke prevention and improving their health consciousness. Increasing public awareness about stroke prevention and planning more frequent physical examinations can enable enhancing the health and well-being of people. 

## Figures and Tables

**Table 1:** Demographics and comorbidity in nurse and control groups.

	Controls (*N* = 334,546)	Nurses (*N* = 83,641)	
	*n* (%)	*n* (%)	*p*-Value
Age, years			0.99
<35	240,170 (71.79)	60,046 (71.79)	
35–50	83,572 (24.98)	20,893 (24.98)	
>50	10,804 (3.23)	2702 (3.23)	
Mean (SD) ^†^	30.28 (9.93)	30.34 (9.70)	0.11
Gender			0.99
Female	332,374 (99.35)	83,098 (99.35)	
Male	2172 (0.65)	543 (0.65)	
Comorbidity ^#^			
Hypertension	691 (0.21)	176 (0.21)	0.83
Diabetes	421 (0.13)	106 (0.13)	0.95
Hyperlipidemia	403 (0.12)	101 (0.12)	0.98
CAD	340 (0.1)	85 (0.1)	0.98
CVD	529 (0.16)	133 (0.16)	0.95
CKD	146 (0.04)	37 (0.04)	0.94
COPD	160 (0.05)	42 (0.05)	0.78
Heart failure	40 (0.01)	10 (0.01)	0.99
Obesity	7 (0)	2 (0)	0.87
Asthma	961 (0.29)	242 (0.29)	0.92
Lung cancer	42 (0.01)	12 (0.01)	0.68

SD—standard deviation; CAD—coronary artery disease; CVD—cardiovascular disease; CKD—chronic kidney disease; COPD—chronic obstructive pulmonary disease. ^#^ Chi-square test; ^†^ *t*-test.

**Table 2:** Incidence rate and hazard ratio for ischemic stroke, stratified with age, sex, and comorbidities.

Variable	Event	P–Y	IR	Crude HR (95% CI)	Adjusted HR ^†^ (95% CI)
Age group, year					
<35	460	3,120,105	0.15	1.00	1.00
35–50	848	1,114,741	0.76	5.15 (4.60–5.77) ***	5.12 (4.57–5.73) ***
>50	629	140,162	4.49	30.5 (27.08–34.44) ***	28.0 (24.77–31.66) ***
Gender					
Female	1921	4,347,072	0.44	1.00	1.00
Male	16	27,936	0.57	1.30 (0.80–2.13)	1.61 (0.98–2.64)
Hypertension					
No	1867	4,366,463	0.43	1.00	1.00
Yes	70	8545	8.19	19.3 (15.2–24.5) ***	1.69 (1.26–2.27) ***
Diabetes					
No	1896	4,369,845	0.43	1.00	1.00
Yes	41	5163	7.94	18.5 (13.6–25.2) ***	2.58 (1.80–3.69) ***
Hyperlipidemia					
No	1905	4,369,902	0.44	1.00	1.00
Yes	32	5106	6.27	14.5 (10.2–20.5) ***	2.57 (1.64–4.04) ***
CAD					
No	1909	4,370,791	0.44	1.00	1.00
Yes	28	4216	6.64	15.3 (10.6–22.3) ***	0.20 (0.07–0.54) **
CVD					
No	1904	4,368,422	0.44	1.00	1.00
Yes	33	6586	5.01	11.6 (8.21–16.3) ***	3.88 (1.57–9.60) **
CKD					
No	1932	4,373,295	0.44	1.00	1.00
Yes	5	1713	2.92	6.70 (2.79–16.12) ***	2.65 (1.08–6.53) *
COPD					
No	1928	4,372,977	0.44	1.00	1.00
Yes	9	2031	4.43	10.1 (5.27–19.5) ***	1.91 (0.93–3.92)
Heart failure					
No	1935	4,374,582	0.44	1.00	1.00
Yes	2	426	4.7	10.9 (2.75–43.3) ***	0.42 (0.10–1.78)
Obesity					
No	1937	4,374,909	0.44	1.00	1.00
Yes	0	--	--	--	--
Asthma					
No	1907	4,362,410	0.44	1.00	1.00
Yes	30	12,598	2.38	5.45 (3.80–7.82) ***	2.37 (1.61–3.50) ***
Lung cancer					
No	1936	4,374,770	0.44	1.00	1.00
Yes	1	238	4.19	10.2 (1.44–72.2) *	2.71 (0.38–19.3)

CAD—coronary artery disease; CVD—cardiovascular disease; CKD—chronic kidney disease; COPD—chronic obstructive pulmonary disease; P–Y—person–years; IR—incidence rate per 1000 person–years; CI—confidence interval; HR—hazard ratio. ^†^ Mutually adjusted with age, sex, and comorbidities using the Cox proportional hazards regression model; * *p* < 0.05, ** *p* < 0.01, *** *p* < 0.001.

**Table 3:** Incidence rate and hazard ratio for hemorrhagic stroke, stratified with age, sex, and comorbidities.

Variable	Event	P–Y	IR	Crude HR (95% CI)	Adjusted HR ^†^ (95% CI)
Age group, year					
<35	254	3,121,312	0.08	1.00	1.00
35–50	368	1,117,173	0.33	4.04 (3.45–4.74) ***	3.96 (3.37–4.64) ***
>50	131	142,368	0.92	11.3 (9.17–13.98) ***	10.02 (8.06–12.5) ***
Gender					
Female	748	4,352,870	0.17	1.00	1.00
Male	5	27,983	0.18	1.04 (0.43–2.51)	1.36 (0.56–3.30)
Hypertension					
No	728	4,372,050	0.17	1.00	1.00
Yes	25	8803	2.84	17.2 (11.5–25.6) ***	2.65 (1.61–4.36) ***
Diabetes					
No	742	4,375,523	0.17	1.00	1.00
Yes	11	5330	2.06	12.2 (6.75–22.2) ***	2.41 (1.22–4.74) *
Hyperlipidemia					
No	747	4,375,608	0.17	1.00	1.00
Yes	6	5245	1.14	11.0 (5.48–22.1) ***	1.33 (0.52–3.39)
CAD					
No	745	4,376,555	0.17	1.00	1.00
Yes	8	4299	1.86	11.0 (5.48–22.1) ***	0.19 (0.06–0.63) **
CVD					
No	739	4,374,172	0.17	1.00	1.00
Yes	14	6681	2.1	12.5 (7.35–21.2) ***	5.95 (2.28–15.5) ***
CKD					
No	745	4,379,140	0.17	1.00	1.00
Yes	8	1713	4.67	27.8 (13.9–55.9) ***	8.00 (3.65–17.5) ***
COPD					
No	752	4378785	0.17	1.00	1.00
Yes	1	2068	0.48	2.82 (0.40–20.1)	1.01 (0.14–7.44)
Heart failure					
No	750	4,380,433	0.17	1.00	1.00
Yes	3	421	7.13	42.5 (13.7–132) ***	1.65 (0.43–6.27)
Obesity					
No	753	4,380,754	0.17	1.00	1.00
Yes	0	--	--	--	--
Asthma					
No	746	4,368,107	0.17	1.00	1.00
Yes	7	12,747	0.55	3.22 (1.53–6.77) **	1.69 (0.78–3.65)
Lung cancer					
No	752	4,380,612	0.17	1.00	1.00
Yes	1	241	4.15	25.6 (3.60–182) **	9.36 (1.32–66.2) *

CAD—coronary artery disease; CVD—cardiovascular disease; CKD—chronic kidney disease; COPD—chronic obstructive pulmonary disease; P–Y—person–years; IR—incidence rate per 1000 person–years; CI—confidence interval; HR—hazard ratio. ^†^ Mutually adjusted with age, sex, and comorbidities using the Cox proportional hazards regression model. * *p* < 0.05, ** *p* < 0.01, *** *p* < 0.001.

**Table 4:** Incidence rate and adjusted hazard ratio for ischemic stroke and hemorrhagic stroke for nurse and control groups, stratified by sex, age, and comorbidity.

	Controls			Nurse			Compared to Control	
Variables	Events	P–Y	IR	Events	P–Y	IR	cHR (95% CI)	aHR ^†^ (95% CI)
Ischemic stroke								
All	1652	3,470,519	0.48	285	904,489	0.32	0.66 (0.58–0.75) ***	0.68 (0.60–0.77) ***
Gender								
Female	1637	3,448,113	0.47	284	898,959	0.32	0.66 (0.59–0.75) ***	0.68 (0.60–0.77) ***
Male	15	22,406	0.67	1	5530	0.18	0.27 (0.04–2.05)	0.29 (0.04–2.17)
Age, years								
<35	358	2,469,514	0.14	102	650,591	0.16	1.08 (0.87–1.34)	1.07 (0.86–1.34)
35–50	741	889,330	0.83	107	225,411	0.47	0.57 (0.47–0.70) ***	0.57 (0.46–0.70) ***
>50	553	111,676	4.95	76	28,486	2.67	0.54 (0.42–0.68) ***	0.54 (0.42–0.68) ***
Comorbidity ^§^								
No	1517	3,441,815	0.44	265	897,073	0.30	0.67 (0.59–0.76) ***	0.68 (0.60–0.78) ***
Yes	135	28,705	4.70	20	7415	2.70	0.57 (0.36–0.92) *	0.58 (0.36–0.93) *
Hemorrhagic stroke								
All	654	3,475,465	0.19	99	905,388	0.11	0.58 (0.47–0.72) ***	0.59 (0.48–0.73) ***
Gender								
Female	650	3,453,020	0.19	98	899,851	0.11	0.58 (0.47–0.71) ***	0.59 (0.47–0.73) ***
Male	4	22,446	0.18	1	5537	0.18	1.01 (0.11–9.00)	1.01 (0.11–9.03)
Age, years								
<35	213	2,470,408	0.09	41	650,904	0.06	0.73 (0.52–1.02)	0.73 (0.52–1.01)
35–50	322	891,471	0.36	46	225,702	0.20	0.56 (0.41–0.77) ***	0.56 (0.41–0.77) ***
>50	119	113,586	1.05	12	28,782	0.42	0.40 (0.22–0.72) **	0.40 (0.22–0.72) **
Comorbidity ^§^								
No	605	3,446,220	0.18	94	897,919	0.10	0.60 (0.48–0.74) ***	0.61 (0.49–0.75) ***
Yes	49	29,246	1.68	5	7469	0.67	0.40 (0.16–1.00)	0.40 (0.16–1.01)

P–Y—person–years; IR—incidence rate per 10,000 person–years; cHR, crude hazard ratio; aHR, adjusted hazard ratio; CI, confidence interval. ^†^ Mutually adjusted with age, sex, and comorbidities. ^§^ Patients with any one of the comorbidities were classified as the comorbidity group. * *p* < 0.05, ** *p* < 0.01, *** *p* < 0.001.
